# PReS-FINAL-2037: Association between clinical and microbiological periodontal condition in Colombian patients with juvenile idiophatic arthritis

**DOI:** 10.1186/1546-0096-11-S2-P50

**Published:** 2013-12-05

**Authors:** C Malagon, C Romero, C Vargas, J De Avila, GI Lafaurie, DM Castillo, AC Mosquera

**Affiliations:** 1 Universidad El Bosque, Bogota, Colombia

## Introduction

Periodontal diseases(PD) are mainly associated with gram negative bacteria that initiate a series of events leading to the loss of periodontal attachment and alveolar bone surrounding teeth. *Porphyromonas gingivalis (Pg) *is considered to be one of the most important oral cavity infectious agents in adults patients. *Pg *is uncommon or found in low numbers in healthy children and don't know information in Juvenile Idiophatic Arthritis (J)IA patients.

## Objectives

To investigate the association between clinical indices of PD and disease activity characteristics in JIA patients receiving treatment.

## Methods

From a rheumatology outpatient clinic 23 JIA patients according to the ILAR classification criteria. Patients with previous periodontal treatment, recent use of antibiotics, infections, less than 10 years and less than 6 teeth, with malignancies or orthodontic treatment were excluded. Demographic, epidemiologic and disease specific variables were collected. Periodontal examination was performed by 2 experienced and calibrated periodontists, assessing bleeding on probing, plaque index, mean and extension of clinical attachment loss, mean pocket depth, inflammation and number of teeth. The extension of periodontitis was evaluated by pocket depth and clinical attachment and the severity of periodontitis was evaluated by clinical attachment loss. *Pg *and *Aggregatibacter actinomycetemcomitans (Aa) *was identified by PCR with species-specific primers from subgingival plaque. The association between clinical index of periodontitis and disease activity measures were analyzed using chi square test, using the SPSS v18 statistics package.

## Results

Twenty three children were included in the study with mean age of 14.7 ± 5.5 years, followed time disease of 78.1 ± 46 months, age of onset 7.7 ± 4.13, painful joints 0.81 ± 1.0, actives joints 0.45 ± 1.0 and limited joints 0.5 ± 1.7. The diagnosis of oligoarticular was establish in 21,7%, poliarticular 34.8%, systemic 13%, enthesitis related arthritis (ERA) 26.1%, Undifferentiated 4.3%, mean of ESR was 11.9 ± 9.7 and CRP 2.5 ± 6.1.HLA-B27 was present in 13.3% of the patients. There was an association between the HLA-B27 and ERA patients (*p = 0.002*).Treatment with methotrexate in 78.8% and anti-TNF in 8.6%. The mean number of teeth was 27.2 ± 1.9, plaque index 3.2% ± 2.4,levels of inflammation(%) were 1.9 ± 2.2 and bleeding on probing 1.9 ± 2.1 and did not present suppuration. The extension and severity periodontitis was clinical acceptable. 34.4% of JIA was positive for *Pg*. *Pg *were observed in 66.7% in ERA patients (*p < 0.05*) and we did not identified Aa.

## Conclusion

These preliminary results shows that treated JIA patients present acceptable periodontal status, the results in ERA JIA for *Pg *was relevant. However, the presence of *Pg *is independent of age, treatment, age of onset, indices of activity and HLA B-27. These results suggest investigate periodontal condition of the family. This is the first study in Colombian children with JIA an PD.

## Disclosure of interest

None declared.

